# Extralinguistic Consultation in English–Chinese Translation: A Study Drawing on Eye-Tracking and Screen-Recording Data

**DOI:** 10.3389/fpsyg.2022.891997

**Published:** 2022-06-20

**Authors:** Yixiao Cui, Binghan Zheng

**Affiliations:** School of Modern Languages and Cultures, Durham University, Durham, United Kingdom

**Keywords:** online consultation, extralinguistic information, English–Chinese translation, eye-tracking, translation experience, translation product

## Abstract

Both linguistic and extralinguistic consultations are essential in translation practice and have been commonly investigated as an integral topic in previous studies. However, since extralinguistic information is usually longer in extent and not specifically designed for a linguistic purpose, extralinguistic consultations involve different search strategies compared with linguistic consultations. Drawing on eye-tracking and screen-recording data, this study compares linguistic and extralinguistic consultations in terms of cognitive resources allocation and information processing patterns in English–Chinese translation. It also explores the differences among 17 language learners, 20 student translators, and 21 professional translators, and the effect of extralinguistic consultation on their translation quality. The findings are as follows: (1) all participants allocate more attention and lower cognitive load to extralinguistic consultations than to linguistic consultations; (2) participants’ translation experience levels and their attention allocated to extralinguistic consultation show an inverted U-shaped relationship; and (3) participants who consult extralinguistic information before drafting or devote more attention to extralinguistic consultation produce target texts with significantly higher scores.

## Introduction

Online consultation behavior in translation can normally involve two types of information from external resources: linguistic information, such as entries in an online dictionary, and extralinguistic information, such as cultural and subject-domain information provided by a general website. Linguistic information is more commonly consulted by translators than extralinguistic information, as is indicated by translators’ greater reliance on using dictionaries (Enríquez Raído, 2014; Zheng, 2014) and a lower frequency of consulting websites (Hvelplund, 2017; Sycz-Opoń, 2019). As a high-quality translation should normally fulfill that “the message embodied in the source text is transferred completely into the target text” (Koby et al., 2014, p. 416), extralinguistic information thus plays an important role in achieving the success of translation (e.g., PACTE, 2003; Gile, 2009; Coban, 2015). Empirical evidence also shows that translators with extralinguistic information on the source text normally produce better translations than those who do not have extralinguistic information (Kim H., 2006). Most existing studies on translation consultation have given more attention to linguistic information than to extralinguistic information (e.g., Atkins and Varantola, 1997; Mackintosh, 1998; Varantola, 1998). Meanwhile, among the few studies that involved extralinguistic consultations, researchers only report the use of extralinguistic references during translating, without describing the detailed consultation behavior or proposing the implied pedagogical suggestions (Hvelplund, 2017; Shih, 2019; Sycz-Opoń, 2019).

Belonging to different information search tasks, linguistic and extralinguistic consultations are triggered by different information needs and involve different search processes. Linguistic consultation is normally triggered by more specific information needs, such as the meaning of a particular term. It belongs to the fact-finding task type, which is defined as “a task in which you are looking for specific facts or pieces of information” (Kellar et al., 2007, p. 1005). Extralinguistic consultation involves the collection of more general information, often from multiple sources and which do not have a specific answer, thus it belongs to the information-gathering task type. Various studies have found that the two types of search tasks are different in required time length and complexity level (Kellar et al., 2007; Cole et al., 2011; Athukorala et al., 2016). In addition, resources for linguistic consultation, such as dictionaries and corpora, are specially designed and ready to use during translation, while resources for extralinguistic consultation, such as web pages and encyclopedias, contain more comprehensive information which needs to be browsed and selected for the translation purpose. The difference in resource designs also affects translators’ consultation behaviors.

Against this background, this study seeks to uncover the nature of extralinguistic consultation and its impact on translation process and product. Eye-tracking (supported by screen-recording) as the main data collection method will be used, as this approach gives “a detailed picture of the complex processing involved in constructing meaning from a string of words or characters and representing that meaning in the words or characters of a new language” (Jakobsen, 2017, p. 33). We aim to address the following research questions: (1) what are the differences between linguistic and extralinguistic consultations? (2) What are the differences in extralinguistic consultation across translators with different experience levels, and how can extralinguistic consultation be optimized? And (3) what are the impacts of extralinguistic consultation on translation quality?

## Research Background

### Linguistic and Extralinguistic Consultations

Behavioral measures and cognitive load have been widely applied in exploring the differences between fact-finding and information-gathering tasks. Behavioral measures include the amount of time spent and the number of pages viewed during information-seeking tasks. Previous studies have consistently found that information-gathering tasks tend to be significantly longer and involve more webpages when compared with fact-finding tasks (Marchionini, 2006; Kellar et al., 2007; Athukorala et al., 2016). Cognitive load is defined as a multidimensional construct representing the load that performing a particular task imposes on a learner’s cognitive system (Paas et al., 1994). Previous studies have reported inconsistent findings regarding cognitive load as indicated by fixation duration allocated to fact-finding and information-gathering tasks. Wang and Zhang (2014) asked participants to conduct one fact-finding task and two information-gathering tasks, and found no significant difference in cognitive load allocation between these two task types. Similarly, Bilal and Gwizdka (2016) studied children’s cognitive load in reading search engine results pages (SERPs) and found no difference in cognitive load between factual tasks (answering a specific question) and research tasks (finding information on a given topic). However, Lu and Jia (2014) suggest that, during image search, participants invested a significantly higher cognitive load in general tasks (searching for broader categories) than in specific tasks (searching for specific objects). Jiang et al. (2014) investigated users’ viewing behavior on the SERPs across four task types: known-item tasks, known-subject tasks, interpretive tasks, and exploratory tasks. They found that users allocated higher cognitive load in the known-item and exploratory tasks than in the known-subject and interpretive tasks. Lewandowski and Kammerer (2020, p. 23) propose an explanation for these inconsistencies, that investigations of the effect of task types on viewing behaviors might be “heavily influenced by the concrete topics, by participants’ familiarity with these topics, and last but not least by the concrete individual search results provided by the search engine.” In the present study, these potential confounders have been well considered. All the participants were asked to translate the same source text with their familiarity to the text background being controlled. We will use both behavioral and cognitive measures to compare linguistic and extralinguistic consultations as two types of information task.

### Translation Experience and Extralinguistic Consultation

Since PACTE (2003) included instrumental sub-competence in its translation competence model, much has been written on improving the use of online resources by comparing translators with different experience levels. These studies have presented two major findings. Firstly, experienced translators show a lower reliance on external resources than inexperienced translators. Kim R. (2006) compared a language learner, a translation student, and a professional translator by analyzing their think-aloud data when translating a financial news article. She found that the language learner relied heavily on reference materials for both known and unknown items, whereas the translation student and the professional translator tended to infer meaning before consulting external resources. Zheng (2014) compared professional translators with novice translators and found that the former made a higher percentage of decisions using predominantly internal support than the latter. Olalla-Soler (2018) reports that, even when student translators already possessed knowledge of a problem, they preferred to double-check by consulting external resources, whereas professional translators tended to rely on their internalized knowledge. These findings can be explained with Pym’s (2015) model of risks and efforts in translation, which suggested that translators might devote different amounts of effort while encountering different levels of risk. When dealing with the need for consulting extralinguistic information, translators’ experience levels might affect their evaluation of the potential risks and thus affect their extralinguistic consultation behaviors.

Secondly, when encountering the need for extralinguistic information, inexperienced translators are prone to consulting linguistic information, while experienced translators tend to seek help from extralinguistic information. Massey and Ehrensberger-Dow (2011, p. 198) reported that “the greatest differences in research behavior between the students and instructors emerged for extralinguistic problems requiring expert or specialized knowledge.” They found that translation students tended to use multilingual online dictionaries, whereas translation instructors consulted more frequently with parallel texts and search engines which provided more adequate information than bilingual dictionaries (Massey and Ehrensberger-Dow, 2011).

In sum, compared with experienced translators, inexperienced translators show a heavier reliance on external resources and prefer to search for extralinguistic information in linguistic resources. It is worth noting that most existing studies have only analyzed the differences in extralinguistic consultation between experienced and inexperienced translators at the text/document level, without giving attention to the analysis of processing units (chunks or segments). Recognizing this limitation, the present study compares the differences in extralinguistic consultation not only at the text level but also at the processing unit level.

### Translation Quality and Extralinguistic Consultation

The effect of extralinguistic consultation on translation quality has been investigated in previous studies from two aspects: (1) the impact of extralinguistic consultation on translation quality and (2) the quantity and quality of extralinguistic information consulted during translation.

Regarding the first aspect, it has been found that translators with extralinguistic knowledge of the source text produce better translation products. Kim H. (2006) divided student translators into two equal groups, with Group 1 being assigned a translation task without any background information, and Group 2 being allowed to collect background information to be used in their translation. She concluded that the Group 2 students produced significantly better target texts. In sight translation and interpreting, the positive effect of extralinguistic consultation as a type of short-term preparation before target language (TL) production has also been observed. Liu (2007) investigated the effect of short-term preparation on the quality of both written translation and interpreting and reports that preparation was positively correlated to students’ performances in both tasks. Zheng and Xiang (2014) assigned 68 interpreting students equally to a control group and an experimental group, with the latter being given relevant cultural background knowledge before a sight translation task. They report that the experimental group produced better target texts than the control group. Song and Tang (2020) compared the interpreting quality produced by students with and without preparation. They found that pre-task preparation could significantly reduce cognitive load of interpreting and improve students’ performance in terminology and logical coherence. In written translation, since translators can consult extralinguistic information during TL production, the research on the impact of the short-term preparation of extralinguistic consultation is neglected. Given these findings, the present study not only investigates the impact on the quality of translations posed by extralinguistic consultations but also compares the quality when extralinguistic information is consulted before and/or during the drafting phase.

Regarding the effect of the quantity and quality of extralinguistic information on translation quality, Kim H. (2006) found that only the quality, but not the quantity, of background information significantly improved the translation scores. However, the experimental design of her study had two major limitations, which may lead to a biased correlated result between the quantity of extralinguistic information and translation quality. Firstly, the participants were required by the researcher, not out of their translation needs, to consult extralinguistic information. Second, it is not an accurate method to calculate the quantity of background information by counting the word number. In the present study, we provide participants with a natural translation environment and use the amount of attention to measure the quantity of extralinguistic information consulted in the task. By devoting their attention, translators focus their conscious awareness on specific environmental stimuli while ignoring other stimuli (Hvelplund, 2021). Therefore, the attention allocation can accurately reflect the quantity of extralinguistic information that has been processed.

To summarize, previous studies have found that translators who have access to extralinguistic knowledge on ST produce significantly better translation products. However, translators who consult a greater amount of extralinguistic information do not necessarily produce better translations. The present study moves a step further by investigating the impact on translation quality from two perspectives: (a) comparing the impact of extralinguistic consultation before and during the drafting phase and (b) improving the method of measuring the information quantity by applying eye-tracking data.

## Materials and Methods

### Participants

Sixty-eight native Mandarin Chinese speakers with English as their second language were recruited as participants on a voluntary basis. None of them had been brought up in a bilingual environment. They were all touch typists and had normal or corrected-to-normal vision. They were told that anonymity and confidentiality would be ensured, asked to sign a consent form, and rewarded with a supermarket gift card. The experiment was approved by the research ethics committee of a United Kingdom University.

Based on their training and work experience, the participants were categorized into three groups: language learners, student translators, and professional translators. The group of language learners (L1 to L22) consisted of 22 participants (20 females and 2 males) with an average age of 21.55 years (range = 20–22, *SD* = 0.78). They were undergraduates in their junior or senior year majoring in English language and literature at Chinese universities with an IELTS overall score ≥7.0. The group of student translators (S1 to S23) consisted of 23 participants (21 females and 2 males) with an average age of 23.45 years (range = 22–28, *SD* = 1.34). They all had an IELTS overall score ≥7.0, finished a 1-year United Kingdom-based MA program in Translation Studies but with no professional translation experience. The group of professional translators (P1 to P23) consisted of 23 participants (12 females and 11 males) with an average age of 44.09 years (range = 38–55, *SD* = 5.15). They were all full-time university lecturers on translation courses, with at least 5 years of freelancing translation experience (mean = 8.73, range = 5–20, *SD* = 4.16) and more than 200,000 words translated. In sum: the language learners had neither translation training nor professional work experience; the student translators only had translation training experience; and the professional translators had both types of experience.

Following Hvelplund (2014), the participants whose eye-tracking data quality satisfied at least two of the following three criteria were included for further analysis: Mean Fixation Duration (MFD) above 200 ms; Gaze Time on Screen (GTS) above 41.34% (one SD below the mean); and Gaze sample to Fixation Percentage (GFP) above 67.52% (one SD below the mean). Nine participants, including five language learners (L1, L16, L19, L20, and L21), three student translators (S1, S10, and S15), and one professional translator (P6), were excluded, with the percentage of invalid data being 13.43%.

### The Material

The experimental text used in this study was an excerpt from an article published in *LiveScience*, a science news website. This text was chosen because the topic was about a Jewish holiday, which was unlikely to be familiar to the participants.

It is worth noting that this study looked into extralinguistic consultations not only in translating the whole text but also in translating selected Rich Points, defined as “specific source text segments that contain translation problems” (PACTE, 2009, p. 214). Two Rich Points were identified from the material based on a pilot study. The first Rich Point “latke” is a type of Jewish food. The TL equivalents, such as “土豆烙饼” or “马铃薯饼” (gloss: pan-fried potato pancake), could be easily located through linguistic consultation. Although being identified as an uncommon word containing a translation problem, this Rich Point was rather straightforward and considered to have a relatively higher need for linguistic consultation and a lower need for extralinguistic consultation. The second Rich Point—“the ‘attendant’ candle” (“头灯” in TL; gloss: headlamp)—refers to the tallest candle used to kindle the other lights in a Jewish menorah. Since no straightforward equivalence in TL could be found through linguistic consultation, this Rich Point was considered to have a relatively higher need for extralinguistic consultation.

### Data Collection

Participants were asked to sit approximately 60 cm away from the monitor and carried out a five-point calibration and validation procedure. After an acceptable calibration had been saved, they started to translate the warm-up text and then the experimental text with no time constraints. They were allowed to use any online resources apart from machine translation and computer-assisted translation (CAT) tools. After the translation task, participants were asked to complete a questionnaire about their educational and professional background and web-searching experience. They were also asked to rank their familiarity with the source text using a five-point Likert scale (1 = “not familiar at all”; 5 = “very familiar”). A ranking of five meant that the participant’s extralinguistic consultation might be influenced by their being “very familiar” with the subject matter, and their data would then be eliminated. In this study, the data from P23 was discarded as the participant reported themself to be very familiar with Jewish culture.

**Figure 1:**
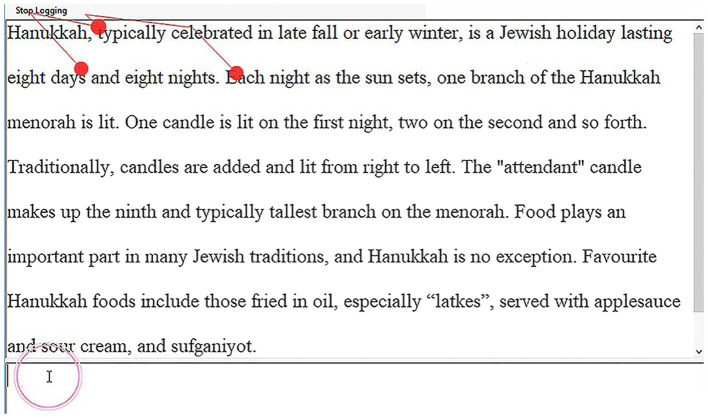
Experimental interface on eye-tracking screen.

All experiments were prepared and run in a lab with an eye-tracker connected to a 23” LCD monitor which functioned as the presentation screen. The screen resolution was set at 1280*1024 pixels, and the fixation radius was the default setting of the Tobii system, 35 pixels per inch. To suit the eye-tracker-based design, we purposely split the screen into two equal areas (shown in Figure 1), with the Translog II user interface on the left for translating and the web browser (Internet Explorer 11) on the right for consultation. The English source text was displayed in the upper window of the Translog interface, with double line spacing and a Microsoft Sans Serif typeface set at 18 points. The Chinese target text was produced in the lower window, with a SimSun typeface set at 18 points, also with double line spacing. The web browser was set up to display a blank page before the translation task began. After each task, the participant’s search history was erased to avoid any potential influence on the next participant.

### Data Annotation

#### Process Data

The process data were collected by eye-tracking and screen-recording methods. Eye-tracking data provided the following three fixation-related measurements.

##### Amount of Attention

This measurement was indicated by total fixation duration (TFD) on a selected Area of Interest (AOI; Jakobsen and Jensen, 2008; Sharmin et al., 2008; Jensen, 2011; Hvelplund, 2019). In the present study, the amounts of attention allocated to linguistic and to extralinguistic consultations were used to evaluate the difference between them from a behavioral aspect: a larger amount of attention meant that the participant spent a longer time on the type of consultation.

##### Cognitive Load

In reading and translation studies, this measurement has commonly been indicated by average fixation duration (Just and Carpenter, 1980; Rayner and Duffy, 1986; Jakobsen and Jensen, 2008; Pavlović and Jensen, 2009; Hvelplund, 2017), with longer fixation duration indicating heavier cognitive load (Hvelplund, 2014). Fixation duration allocated to linguistic and extralinguistic consultations was used to evaluate their difference in cognitive load.

##### Viewing Pattern

This measurement refers to the ways in which participants scan and read the webpages under consultation, and is reflected in two visualization tools: gaze plots and heat maps (Simonsen, 2011; Gossen et al., 2014a,b). Gaze plots show the location, order, and time spent looking at locations on the stimulus and are used to reveal the time sequence of fixations, while heat maps show how the fixations were distributed over the stimulus (Tobii Pro, 2021).

Screen-recording videos were transcribed for further investigation with the aim of creating a script “resembling ‘stage directions’” (Pavlović, 2007, p. 76) and detailing each information search behavior. To this end, we adopted Hargittai’s (2004, p. 210) transcription method for coding and classifying users’ online information-seeking behavior. This method contains three key components: (a) an online action, such as accessing a uniform resource locator (URL), clicking on a button or a link, or searching; (b) specific details of the action, such as the URL, the timestamp, and the search query; and (c) a search result, such as whether the search was successful or not. We did not follow this method completely since it was developed to describe general online information-seeking behaviors and “not all projects [might] require the level of detail” (Hargittai, 2004, p. 211). Instead, we selected some of the relevant components and proposed a transcription convention consisting of the timestamp, online action, query sequence, URL, search query, and type of information on the visited webpage, the latter which was categorized as linguistic information (e.g., entries in dictionaries) or extralinguistic information (e.g., encyclopaedical contents). Table 1 presents a transcription example of (S5) screen-recording data, which shows that this search episode contained three queries: one linguistic consultation and two subsequent extralinguistic consultations.

**Table 1:** A transcription example (S5) of screen-recording data.

Time	Action	Query sequence	URL	Search query	Information type
07.46	Search	1	Dictionary.com	Attendant	Linguistic
	Evaluate				
08.18	Search	2	En.wikipedia	Hannukah	Extralinguistic
	Read the result page with highlight				
11.30	Search	3	Google	Shamash	Extralinguistic
	Evaluate				

#### Product Data

Product data include the quality assessments of the target texts. In the past two decades, a variety of assessment methods have been developed and applied in translation teaching and practice, such as calibration of dichotomous items (Eyckmans and Anckaert, 2017), comparative judgment (Han, 2020). When narrowing down to the studies on the effect of consultation on translation quality, we found that the error-based assessment method (PACTE, 2003; Liu, 2007) and the holistic judgment method (Kim H., 2006; Zheng, 2014) have been widely used.

**Figure 2:**
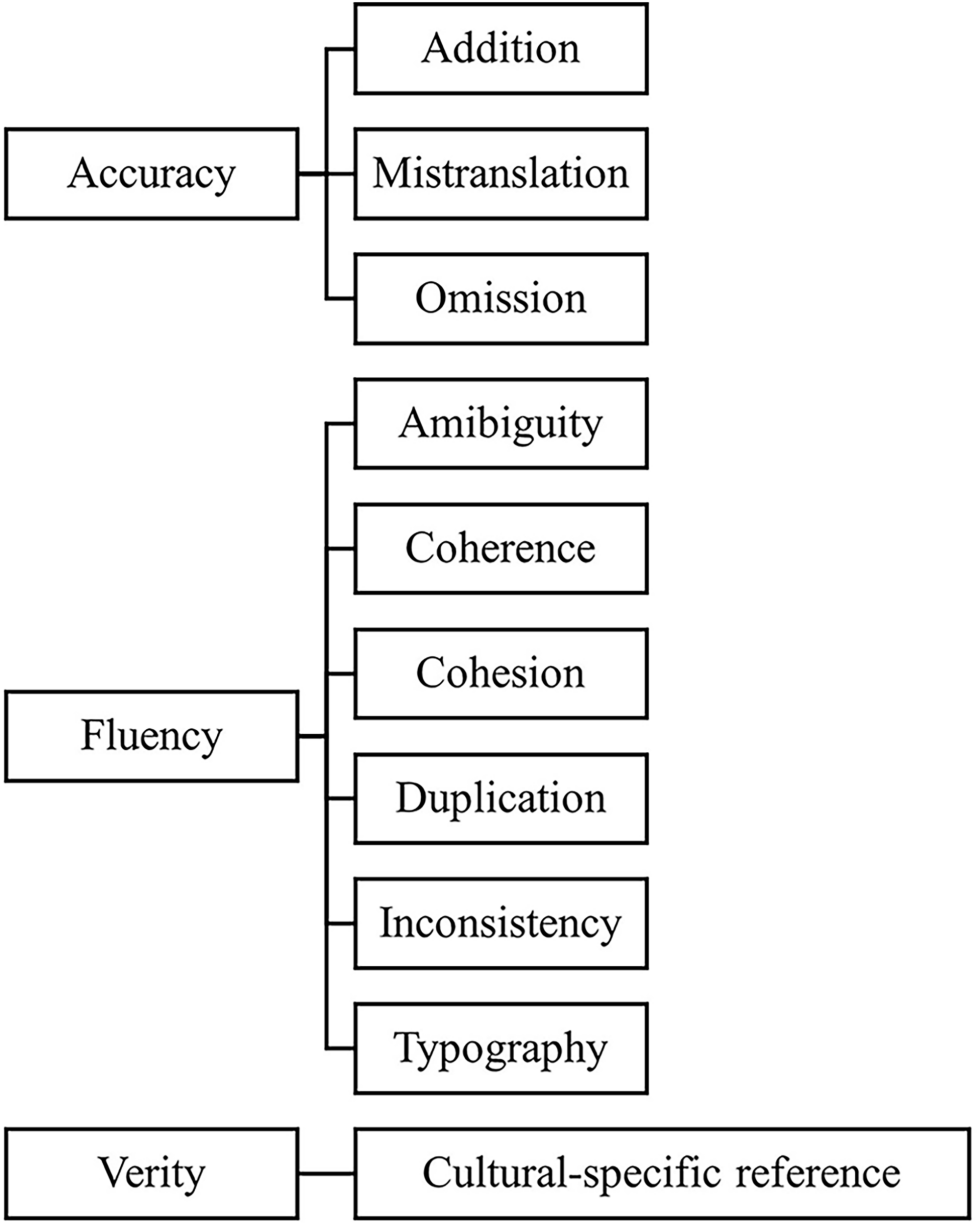
Refined Multidimensional Quality Metrics (MQM) metrics applied in this study.

In the present study, we used the Multidimensional Quality Metrics (MQM) framework. Developed by the QT Launchpad project (Burchardt and Lommel, 2014), this framework provides a flexible vocabulary of quality issue types and a mechanism for applying them to generate quality scores (Klubička et al., 2017). It does not impose a single metric for all uses but includes a comprehensive catalog including 108 quality issue types that can be categorized into five subdivisions: fluency, accuracy, verity, design, and internationalization (Lommel et al., 2013). As the task in the present research was translating from scratch without using CATs, we selected 10 metrics from three subdivisions: fluency, accuracy, and verity (see Figure 2).

Two professional translators were invited to assess all target texts. They had prior experience of using the MQM framework and were thoroughly familiarized with the official guidelines and assessing procedure. Each of them was given a portfolio which includes the source text, a reference translation, and 58 target texts. They were asked to identify problematic issues into categories of accuracy penalty (AP), fluency penalty (FP), or verity penalties (VP), and to annotate them as minor, major or critical.

After receiving all the evaluation reports, we calculated the penalty points and, subsequently, the scores of translation quality (TQ), using the following formulae (Lommel et al., 2013, p. 6):


(1) $$\mathrm{Penalty}=\left({\mathrm{Issue}}_{\mathrm{minor}}+{\mathrm{Issue}}_{\mathrm{major}}\times 5+{\mathrm{Issue}}_{\mathrm{critical}}\times 10\right)÷\mathrm{Word Count}$$

(2) TQ=100−AP−FP−VP

Fleiss’ kappa (Fleiss, 1971) was calculated to measure the inter-rater reliability of the TQ scores. The resulting kappa score was 0.875 and *p* < 0.001, indicating an almost perfect agreement between the raters (Landis and Koch, 1977). The mean value of translation scores was used as the final score for each target text. The statistical results of the translation scores for each group of participants are presented in Table 2. As the translation experience increases, the translation scores show a significant upward tendency.

**Table 2:** Statistical results of translation scores for the three groups.

Group	*N*	Mean score	*SD*	ANOVA	
Language learners	17	66.06	12.21	*F* = 6.240	*p* < 0.05
Student translators	20	71.92	13.71		
Professional translators	21	80.72	12.71		

## Results

### Differences Between Linguistic and Extralinguistic Consultations

When translating the entire text, the differences between linguistic and extralinguistic consultations were explored from three perspectives: (a) the amount of attention indicated by TFD; (b) cognitive load indicated by average fixation duration; and (c) the reading path reflected in heat maps and gaze plots.

**Figure 3:**
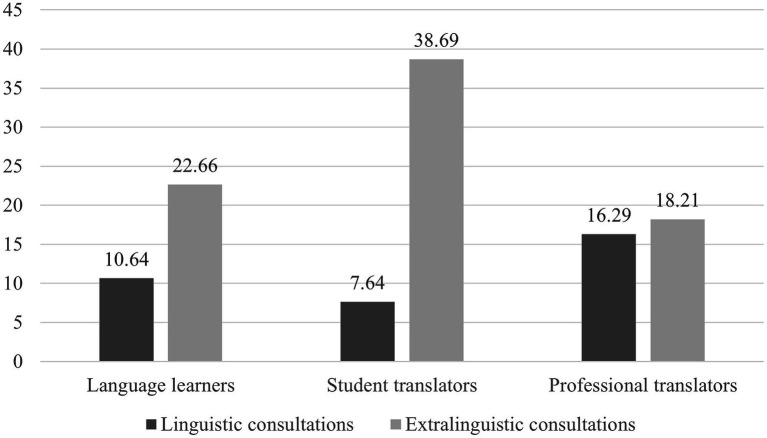
Mean total fixation duration (TFD; in seconds) on linguistic and extralinguistic consultations.

As can be seen from Figure 3, the mean TFDs on extralinguistic consultations were greater than on linguistic consultations for all three groups. Paired *t*-tests were utilized for the comparison “in which the same participants are tested at two different times or for two different treatments” (Mellinger and Hanson, 2017, p. 104). The results show that the differences were statistically significant for language learners [*t*(16) = 1.597, *p* < 0.05, *d* = 0.81] and student translators [*t*(19) = 4.042, *p* < 0.001, *d* = 0.65], but not for professional translators [*t*(20) = 0.458, *p* > 0.05].

**Figure 4:**
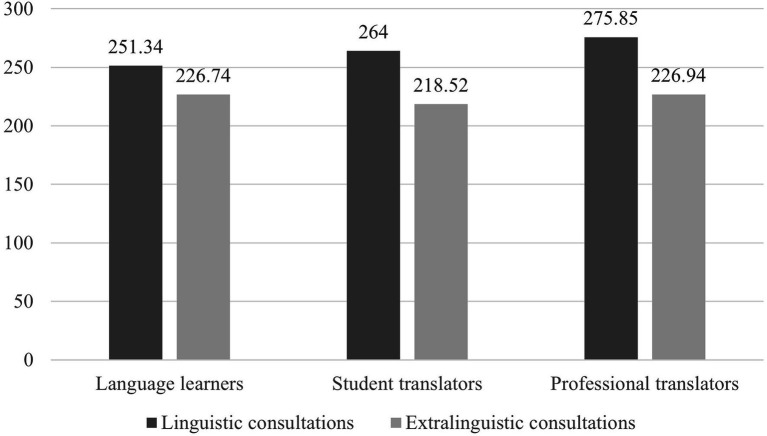
Average scores of fixation duration (in milliseconds) allocated to linguistic and extralinguistic consultations across three groups.

Regarding cognitive load, Figure 4 presents the average scores of fixation duration allocated to linguistic and extralinguistic consultations by each group of participants. It shows that all participants allocated higher cognitive load to linguistic consultations than to extralinguistic consultations. Paired *t*-tests confirm that the differences were statistically significant for all three groups [*t*(16) = 1.283, *p* < 0.05. *d* = 0.80 for language learners; *t*(19) = 4.210, *p* < 0.001, *d* = 0.81 for student translators; and *t*(20) = 4.402, *p* < 0.001, *d* = 0.67 for professional translators].

As for viewing patterns, we examined the heat maps and gaze plots of the linguistic and extralinguistic consultations. In general, for linguistic consultations, regardless of their experience levels, the participants focused on target pages from online dictionaries or the lexical information sections from search engines. Their fixations formed a horizontal path and were concentrated on the relevant information, which was located in a small area of the webpage. Whereas for extralinguistic consultations, the participants tended to read SERPs, content from encyclopedias, or relevant information portals. Their fixations were scattered over a larger area including more information, and followed a vertical path.

In summary, linguistic and extralinguistic consultations possess the characteristics of fact-finding and information-gathering tasks, respectively. Compared with linguistic consultations, extralinguistic consultations attracted significantly larger amounts of attention, and required lower cognitive load and more scanning.

### Extralinguistic Consultations Across Participant Groups With Different Experience Levels

In this section, we will explore the differences in extralinguistic consultations across the three groups of participants from two levels: (a) when they translate the entire text and (b) when they translate selected Rich Points. It is important to note that a comparison between the language learners and student translators, and a comparison between the student and professional translators, are carried out separately. This is because the language learners and the student translators mainly differ in their training experience, while the student and professional translators in their professional experience. The language learners and professional translators are different in both aspects of translation experience, training, and work experience, so that the comparison between these two groups is not considered to be reliable.

#### Differences When Translating the Entire Text

**Figure 5:**
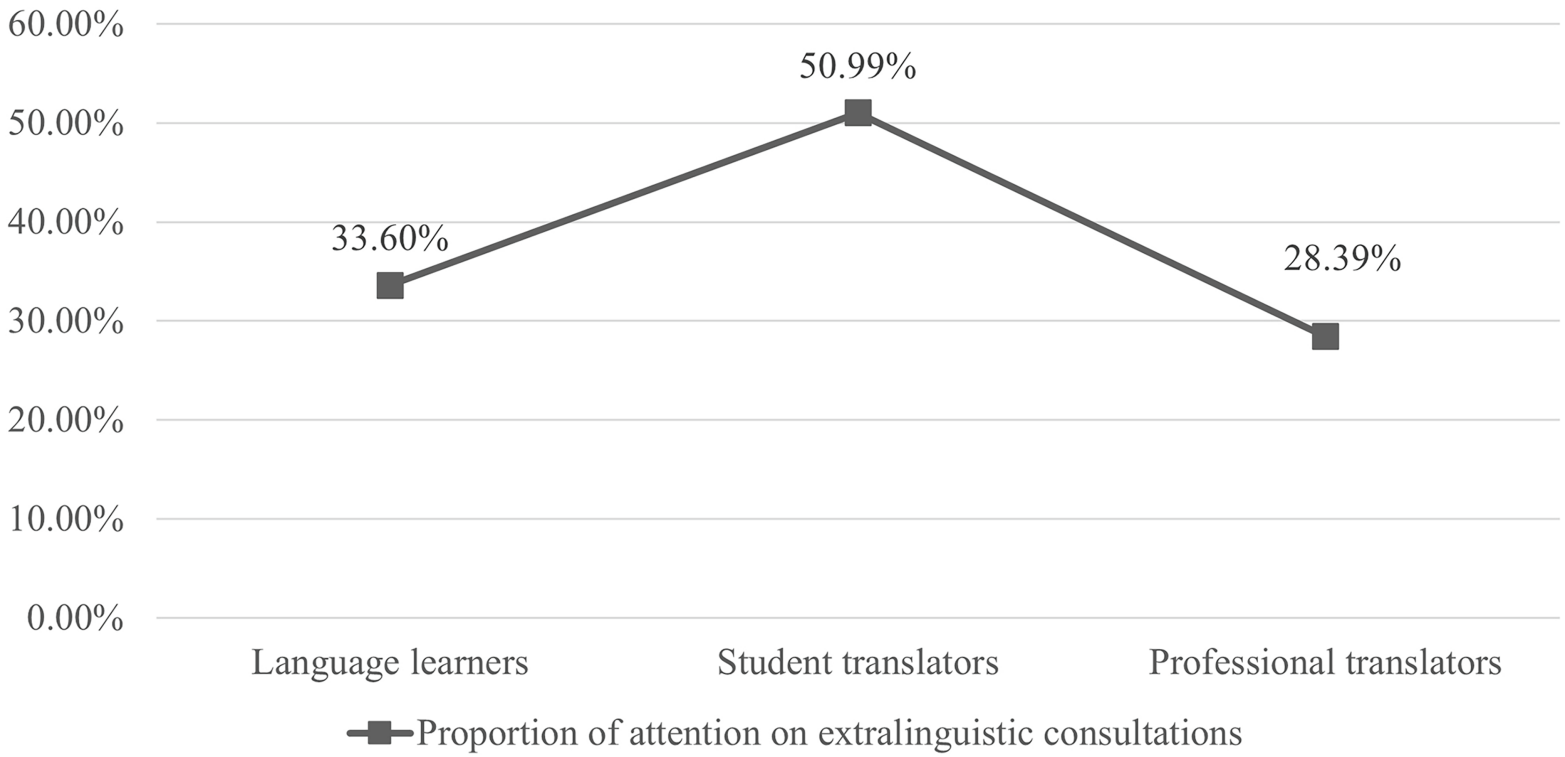
Mean proportion of TFD allocated to extralinguistic consultations.

When translating the entire text, the reliance on extralinguistic consultations was indicated by the proportion of TFD allocated to extralinguistic consultations over the amounts of TFD on the entire consultation process (see Figure 5). Among three groups of participants, student translators allocated the largest proportion of attention to extralinguistic consultations. Independent *t*-tests were the parametric tests to determine “whether the means of the two independently measured groups differ at a statistically significant level” (Mellinger and Hanson, 2017, p. 91). The results show that the difference between the language learners and the student translators was not statistically significant [*t*(35) = −1.860, *p* > 0.05], but the difference between the student and professional translators was statistically significant [*t*(39) = 2.697, *p* < 0.05, *d* = 0.82].

Another difference at the text level was whether the participants performed a pre-translation preparation, which was reflected in the timestamp associated with the extralinguistic consultations. Jakobsen (2002) divided the cognitive processes of translation into three stages: the orientation stage, the drafting stage, and the end revision and monitoring stage. The drafting stage begins with the typing of the first text production keystrokes and ends when the translation of the last sentence is completed. Table 3 presents the number and percentage of participants in each group with or without preparation before the drafting stage. Compared with the language learners and professional translators, more student translators had short-term preparation with extralinguistic information before drafting.

**Table 3:** Number (and percentage) of participants with or without a short-term preparation before drafting.

Group	With preparation	Without preparation
Language learners	2 (11.76%)	15 (88.24%)
Student translators	8 (40.00%)	12 (60.00%)
Professional translators	6 (28.57%)	15 (71.43%)

For those participants who conducted preparation before drafting, they followed almost the same procedure: read through the source text and then use search engines to find subject information. But for those without preparation, we noticed an intriguing difference across the three groups. A large number of language learners (13/15, 86.67%) and student translators (10/12, 83.33%) who did not conduct pre-translation preparation consulted extralinguistic information during drafting. However, this kind of postponed extralinguistic consultation was rarely seen among the professional translators (7/15, 46.67%).

#### Differences When Translating Rich Points

We further compared the differences across the three groups of participants in extralinguistic consultations when translating selected Rich Points. The investigation was based on two metrics: (a) the proportion of attention (indicated by TFD) distributed to extralinguistic consultations over the amount of attention distributed to the entire consultation process and (b) translators’ consultation styles and their preference toward linguistic or extralinguistic consultations.

**Figure 6:**
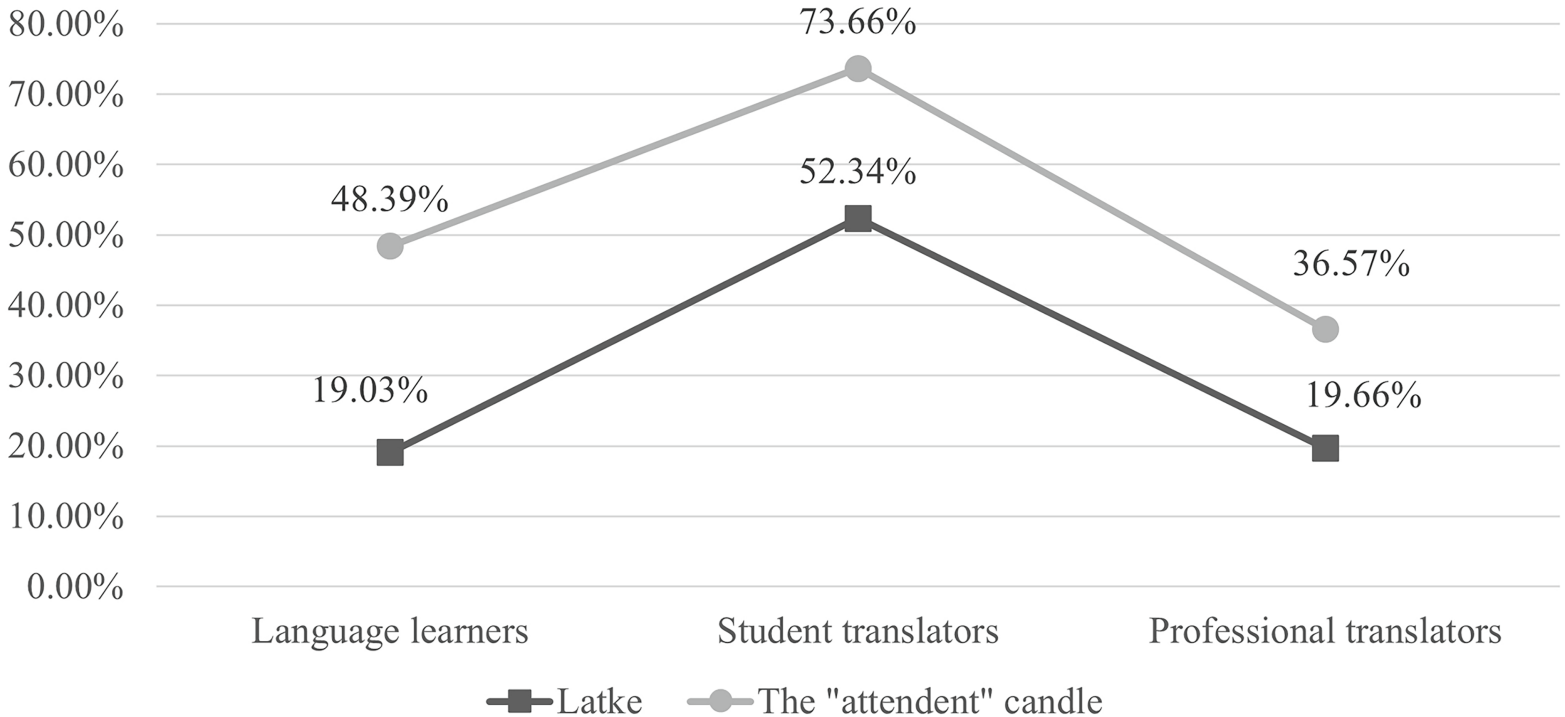
Mean proportions of TFD allocated to extralinguistic consultations when translating two Rich Points.

Figure 6 presents the mean proportions of TFD allocated to extralinguistic consultations when translating the two Rich Points. Firstly, among all participants, a larger proportion of attention was devoted to extralinguistic consultations on “latke” than on “the ‘attendant’ candle.” Secondly, the participants’ translation experience and the proportion of attention allocated to extralinguistic consultation displayed a non-linear, inverted U-shaped relationship. In other words, the student translators (moderately experienced translators) devoted a larger proportion of attention than both the language learners (the least experienced translators) and the professional translators (the most experienced translators).

The results of independent *t*-tests (Table 4) show that for “latke,” both between-group differences were statistically significant; whereas for “the ‘attendant’ candle,” a significant difference was only shown between student and professional translators.

**Table 4:** Between-group comparisons for attention distribution when translating two Rich Points.

Comparison group	Latke				The “attendant” candle			
	*t*	*df*	*p*	*d*	*t*	*df*	*p*	*d*
Language learners vs. student translators	2.230	35	<0.05	0.65	1.842	28	>0.05	/
Student translators vs. professional translators	−2.355	39	<0.05	0.77	−3.076	32	<0.05	0.80

The second aspect of the differences when translating Rich Points was the translators’ consultation styles, which were analyzed based on transcriptions of the screen-recording videos. When translating “latke,” all translators’ initial consultation was on linguistic information. However, a different searching style was found in their subsequent extralinguistic consultations. Student translators tended to reconfirm the initial search result with further extralinguistic consultations even after finding an acceptable TL equivalent. However, language learners and professional translators seldom had a reconfirmation search: when they found an acceptable lexical meaning through linguistic consultations, they returned to translating the following part of the text.

When translating “the ‘attendant’ candle,” the translators also started their consultations with linguistic information. In this case, however, they failed to locate any acceptable TL equivalent. When language learners could no longer locate TL equivalents, they prolonged their consultations by searching for extralinguistic information, leading to the increase in the proportion of attention devoted to extralinguistic consultations when translating the two Rich Points (19.02% and 48.39%). Student and professional translators, on the other hand, did not change their consultation style much when translating this Rich Point. Student translators always sought help from extralinguistic information no matter whether linguistic consultations were successful or not, whereas professional translators tended to rely on their internal knowledge when they failed with linguistic consultations.

### Effect of Extralinguistic Consultation on Translation Quality

The effect of extralinguistic consultation on translation quality was investigated from three perspectives: (a) comparing the quality with or without preparation before drafting; (b) comparing the quality with pre-task preparation or with consultation during drafting; and (c) examining the correlations between the quantity of extralinguistic information consulted (indicated by the amount of TFD) and translation quality.

**Figure 7:**
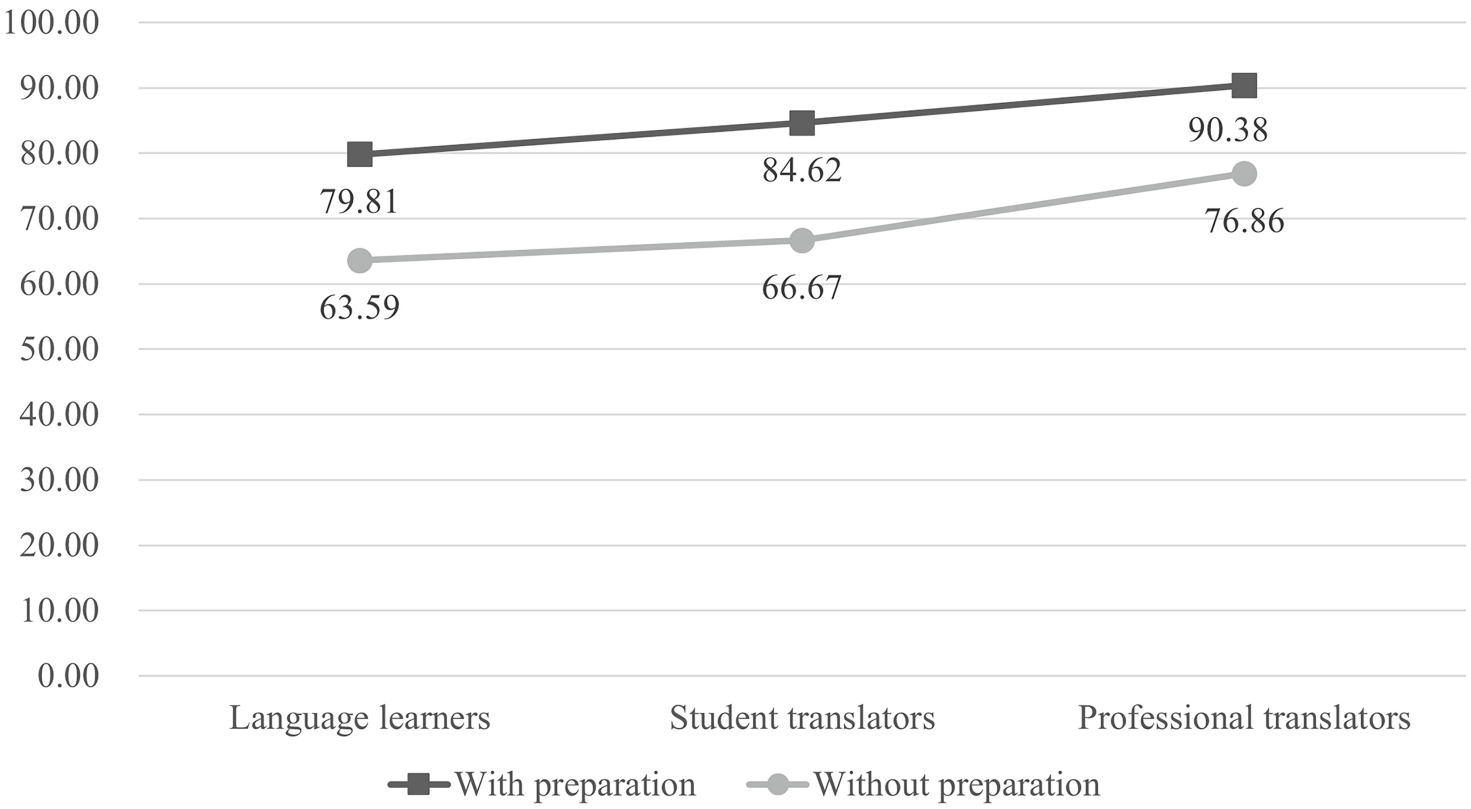
Mean translation quality scores for each group of participants with or without preparation.

Figure 7 presents the mean quality scores for each group of participants with and without preparation. It suggests that, regardless of their experience levels, participants who prepared by extralinguistic consultation before drafting produced better translation results. Independent *t*-tests show that the differences were statistically significant [*t*(15) = 2.700, *p* < 0.05, *d* = 0.81 for language learners; *t*(18) = 2.333, *p* < 0.05, *d* = 0.77 for student translators; and *t*(19) = 2.466, *p* < 0.05, *d* = 0.78 for professional translators].

**Figure 8:**
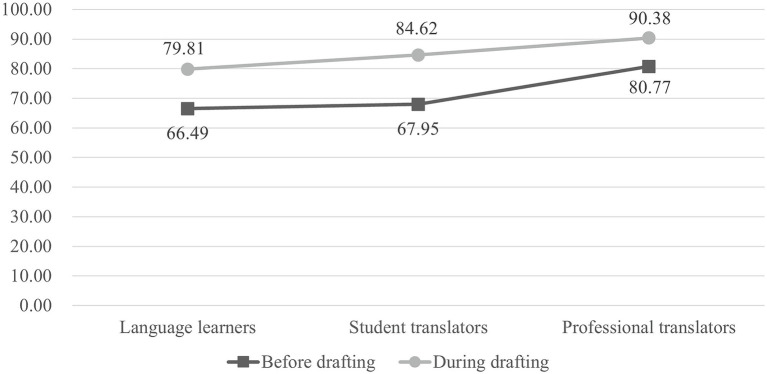
Mean translation quality scores for participants with extralinguistic consultations before and during drafting.

We then compared the translation scores of the participants who had extralinguistic consultations before and during drafting (see Figure 8). The results show that the participants who consulted before drafting performed significantly better than those who consulted during drafting [*t*(13) = 3.098, *p* < 0.05, *d* = 0.80 for language learners; *t*(16) = 1.589, *p* < 0.05, *d* = 0.67 for student translators; and *t*(11) = 2.568, *p* < 0.05, *d* = 0.78 for professional translators].

The Spearman correlation coefficients were conducted to measure the strength of the linear relationship between the amount of attention allocated to extralinguistic consultations and translation quality (Mellinger and Hanson, 2017). The results are presented in Table 5, revealing statistically significant relationships for all groups of participants.

**Table 5:** Spearman correlation coefficients between the amount of attention allocated to extralinguistic consultations and translation quality.

Group	Amount of attention	
	*ρ*	*p*
Language learners	0.693	<0.05
Student translators	0.513	<0.05
Professional translators	0.740	<0.001

## Discussion

### Linguistic and Extralinguistic Consultations

Our results confirm that linguistic and extralinguistic consultations possess the characteristics of fact-finding and information-gathering tasks, respectively. Three differences between these two types of consultation can be summarized as follows. Firstly, translators allocate a larger amount of attention to extralinguistic consultations than to linguistic consultations. This difference is in line with previous findings on general information tasks: information-gathering tasks take longer to accomplish than fact-finding tasks (Marchionini, 2006; Kellar et al., 2007; Cole et al., 2011; Athukorala et al., 2016). Secondly, translators devote lower cognitive load to extralinguistic consultations than to linguistic consultations. This suggests that linguistic and extralinguistic consultations involve different information processing patterns. Brand-Gruwel et al. (2009) proposed the “Information Problem Solving on the Internet” model to classify Internet information processing patterns. The five main processing patterns were problem definition, searching, scanning information, deep processing, and presentation. In linguistic consultations, the relevant information is usually placed compactly and is easier to process without much scanning. In extralinguistic consultations, however, the pertinent information is normally included on a lengthy webpage and requires scanning to be located. The third difference can be observed in the viewing patterns drawn by gaze plots and heat maps, which show that translators tend to conduct more scanning and less deep processing when consulting extralinguistic information.

Based on these findings, we propose two implications for translation pedagogy and further research. Firstly, translation teachers should consider ways of improving the efficiency of extralinguistic consultations. For example, they could demonstrate the use of built-in search functions, which could highlight keywords on the SERPs and accelerate the scanning process. Secondly, since linguistic and extralinguistic consultations reveal different characteristics and require different search techniques, further studies should consider extralinguistic consultation as a sole research object rather than an integral part of consultation, for an optimal result.

### Translation Experience and Extralinguistic Consultation

In translating an entire text, two differences in extralinguistic consultations are found across three groups of translators. Firstly, translators’ experience levels and the proportion of attention allocated to extralinguistic consultations forms an inverted U-shaped relationship: language learners devote insignificantly less attention to extralinguistic consultation than student translators, while professional translators have a significantly lower reliance on extralinguistic consultation than student translators. This finding is consistent with Olalla-Soler (2018), who reports an increase in both the number of queries and in time spent on consultations following an increase in translation training time, but less reliance on consultations from translation students to professional translators. Moorthy et al. (1997) also note this relationship, between consumers’ shopping experience and the amount of external search, arguing that consumers’ experiences affect their search behaviors in two phases: consumer expertise is initially dominant, leading to a greater need for information; but the knowledge effect takes over beyond a certain level of experience, leading to a decline in the curve. This argument can be applied to explain the relationship between translators’ experience levels and their reliance on extralinguistic consultations. On the one hand, compared with language learners, student translators “are trained intensively and systematically in how to recognize what specific information needs they have with regard to a given translation assignment and how to fulfil that need” (Kastberg, 2002, p. 62), so they are more proficient in using external resources. Nevertheless, although language learners “are not aware of the possibilities offered by the different instrumental resource types” (Kuznik and Olalla-Soler, 2018, p. 23), they still know how to use online resources. Kuznik and Olalla-Soler (2018) also report that first-year students, who do not have any translation experience, are capable of using external resources. On the other hand, student translators tend to use information resources without establishing their information needs or planning queries (Enríquez Raído, 2014; Olalla-Soler, 2018), whereas professional translators have a lower reliance on external resources (Fraser, 2000; House, 2000; Zheng, 2014).

Another difference reported in the present study relates to translators’ consultation styles regarding how they search for subject knowledge. Non-professionals (language learners and student translators) who do not consult extralinguistic information before drafting tend to search for extralinguistic information during drafting, whereas professional translators without extralinguistic consultation before drafting do not conduct similar behaviors. Sirén and Hakkarainen (2002, p. 73) point out that, “before solving a problem, experts may spend time analyzing it, while novices often attempt to solve a problem immediately.” Even when novices have started drafting without any extralinguistic consultation, this does not necessarily mean that they have sufficient knowledge to translate the text, which is reflected in their postponed consultations.

In translating selected Rich Points, translators with different experience levels perform different styles of extralinguistic consultation. Pym’s (2015) model of risks and efforts can be used to explain this situation (see Table 6).

**Table 6:** Pym’s (2015) model of risks and efforts.

	Low chance of non-cooperation	High chance of non-cooperation
Low effort	Low-risk (risk aversion, risk transfer)	Mid-risk (“under-work,” guesswork)
High effort	Mid-risk (overwork, inefficient labor)	High-risk (risk transfer, risk taking)

Based on this model, translating “latke” and “the ‘attendant’ candle” without extralinguistic consultation could be considered as two different tasks: one with a low chance of non-cooperation, and the other with a high chance of non-cooperation. Facing the difference in uncertainty between the two tasks, student translators and professional translators exercise the same level of effort. Students always devote a high level of effort even though this may be inefficient labor; whereas professionals, who are more confident in their internal knowledge, devote a lower level of effort to consulting external information. This finding is in line with Künzli (2004) who reported that experienced translators are more likely to use risk transfer strategies than inexperienced translators. Fraser (2000) and House (2000) also pointed out that professional translators are high-risk takers and have a lower reliance on external resources when compared with non-professionals.

### Translation Quality and Extralinguistic Consultation

This study shows that translators who prepare with extralinguistic knowledge before drafting produce significantly better target texts. This finding is consistent with Kim H. (2006) and Liu (2007), both of whom have reported a positive correlation between the availability of extralinguistic knowledge on translation quality. Apart from the fact that sufficient subject knowledge is key to developing translation competence (PACTE, 2003), we also consider extralinguistic consultations before drafting as a type of pre-task planning. By conducting the action of planning, translators postpone the drafting stage and prepare themselves for potential problems. Various studies have reported that planning can benefit task performance. For example, Unterrainer and Owen (2006) reported that, when participants were instructed to plan ahead well before starting to interact with the task, their performance would be improved. Furthermore, Albert and Steinberg (2011) used first move latency to indicate the length of initial planning phase in a problem-solving task and found that first move latency is positively related to task performance. In a translation process that involves many problem-solving tasks, extralinguistic consultations before drafting enable translators to have an all-sided planning of the task, which will have positive effect on the translation performance.

The present research also reports that translators who consult a greater amount of extralinguistic information produce better target texts. This differs to the finding reported by Kim H. (2006) who found that the quantity of background information had little effect on translation quality. Compared with her research, this study has improved the method of calculating information quantity by using the amount of attention, which results in the different findings. In this study, since all the participants did not have any background knowledge about the source text, extralinguistic consultations became the only method of enhancing their domain knowledge. Previous studies have found that web search contributes to an increase in domain knowledge, from two perspectives: (a) comprehending the question (Foltz, 1996) and (b) subsequent information acquisition (Desjarlais and Willoughby, 2007; Lawless et al., 2007). In translation, extralinguistic consultations could benefit the production of target texts likewise from these two perspectives. Translators who consult a greater quantity of extralinguistic information have a better understanding of the source text, which contributes to the production of target texts. In addition, when translators consult extralinguistic information before drafting, they prepare themselves with more domain knowledge and thus “are expected to utilize more appropriate terms in the queries and to know more terms (synonyms) related to the topic” (Aula, 2005, p. 26).

## Conclusion

Using a combination of eye-tracking and screen-recording data, this study uncovered the nature of extralinguistic consultation by exploring the differences between linguistic and extralinguistic consultations. It further provided pedagogical suggestions for translation training by comparing extralinguistic consultation behaviors across translators with different experience levels and through investigating the effect of extralinguistic consultation on translation quality. In summary, this study shows that: (a) translators, irrespective of their translation experience, allocate a larger amount of attention, a lower cognitive load, more scanning and less deep processing to extralinguistic consultations than to linguistic consultations; (b) translators’ experiences and the proportion of attention allocated to extralinguistic consultations forms an inverted U-shaped relationship; (c) non-professionals who do not conduct pre-translation preparation tend to consult extralinguistic information during drafting, whereas professional translators rarely conduct extralinguistic consultation during drafting phase; (d) translators with different experience levels adopt different risk management strategies: non-experts have a heavier reliance on external information and tend to confirm search outcomes by consulting multiple information sources; while professional translators have a lower level of dependency on extralinguistic information and prefer solving problems with their internal knowledge; and (e) among translators with the same level of experience, those who prepare with subject knowledge before drafting or who devote more attention to extralinguistic consultations produce target texts with significantly higher scores.

Our research evidences the importance of extralinguistic consultation in producing high-quality translation and provides implications for improvements in translation pedagogy and for future research. We suggest that pre-task preparation, which has been widely studied in sight translation and interpreting, should receive more attention in written translation from translation instructors and researchers.

This study has certain limitations since it has been conducted using one source text under one experimental environment. Consequently, this study could be replicated with multiple specialized source texts, such as legal documents and medical reports, and with more language directions. It could be designed as a longitudinal study on translation students regarding the development of their extralinguistic consultation skills. In this way, it would be possible to (a) collect data on more diverse extralinguistic consultations; (b) investigate how the unique linguistic features of Mandarin could have an effect on translators’ consultation behaviors; and (c) provide more suggestions for translation pedagogy based on translation students’ attitude toward the training.

## Data Availability Statement

The raw data supporting the conclusions of this article will be made available by the authors, without undue reservation.

## Ethics Statement

The studies involving human participants were reviewed and approved by the ethics committee of Durham University. The patients/participants provided their written informed consent to participate in this study.

## Author Contributions

YC designed the study, collected, annotated, and analyzed the data, and wrote the original draft. BZ conceptualized and designed the study, reviewed and edited the manuscript, and supervised the project. The two authors contributed equally to the article and approved the submitted version.

## Funding

The work was supported by the National Social Science Fund of China (no. 20BYY014). YC is funded by the China Scholarship Council (CSC no. 201808060310).

## Conflict of Interest

The authors declare that the research was conducted in the absence of any commercial or financial relationships that could be construed as a potential conflict of interest.

## Publisher’s Note

All claims expressed in this article are solely those of the authors and do not necessarily represent those of their affiliated organizations, or those of the publisher, the editors and the reviewers. Any product that may be evaluated in this article, or claim that may be made by its manufacturer, is not guaranteed or endorsed by the publisher.
